# A Comprehensive Literature Review and Case Report of Severe Lymphoproliferative Disease Secondary to *CD137* Deficiency

**DOI:** 10.3390/jcm15114291

**Published:** 2026-06-01

**Authors:** Abeer S. Algrafi, Turki Alwasaidi, Mohammed Albalawi, Mohsen Alzahrani, Saad Almutairi, Haitham Osman

**Affiliations:** 1 Department of Internal Medicine, College of Medicine, Taibah University, Madinah 42353, Saudi Arabia; 2 Department of Medicine, Prince Mohammed Bin Abdulaziz Hospital, Ministry of National Guard Health Affairs, Madinah 42324, Saudi Arabia; 3 Department of Oncology, King Abdulaziz Medical City, Ministry of National Guard Health Affairs, Riyadh 11426, Saudi Arabia

**Keywords:** primary immunodeficiency disorders, *CD137* deficiency, *TNFRSF9* mutations, Epstein–Barr virus (EBV), lymphoproliferative disease

## Abstract

Inborn errors of immunity, including primary immunodeficiency disorders (PIDs), comprise a heterogeneous group of genetic conditions characterized by immune system dysfunction. One such rare PID is *CD137* deficiency, which results from *TNFRSF9* mutations. *CD137*, also known as 4-1BB, plays a pivotal role in immune system regulation and co-stimulation. This literature review explores *CD137* deficiency and its implications, emphasizing its association with EBV-associated lymphoproliferative disease and potential therapeutic targets. We present the case of a 21-year-old female patient with *CD137* deficiency who experienced recurrent infections, autoimmunity, and lymphoma. Genetic analysis revealed that the patient had a homozygous *TNFRSF9* variant. The patient subsequently developed severe Epstein–Barr virus (EBV)-associated lymphoproliferative disease, which is one of the clinical manifestations associated with *CD137* deficiency. Additionally, this review discusses similar cases in the literature and details the clinical manifestations and immune abnormalities associated with *CD137* deficiency. Understanding the genetic complexity of *CD137* deficiency and the immune system dysregulation it causes provides insights into potential therapeutic interventions for affected individuals. This review highlights the role of *CD137* as a crucial regulator of immune homeostasis and a potential target for immunotherapy in autoimmune diseases and malignancies.

## 1. Introduction

Inborn errors of immunity (IEIs) are a heterogeneous group of disorders characterized by impaired immune function, predisposing affected individuals to infections, autoimmunity, inflammation, and malignancy [1]. To date, 508 IEIs have been described, and new findings of genetic defects keep advancing our knowledge of the immunological and molecular processes involved in human immune diseases [2]. IEI-related primary immunodeficiencies and immune dysregulatory disorders are rare. Global registry data indicate that the prevalence of primary immunodeficiency disorders varies widely among different populations, ranging from approximately 1 in 8500 to 1 in 100,000 symptomatic individuals [3].

In contrast to recurrent infections, IEIs are now known to involve a variety of syndromes that are predominantly characterized by immunological dysregulation and autoimmunity, with up to 25% of patients having an increased risk of cancer [4]. A study of 35 patients with PIDs reported that the most common lymphoproliferative diseases and malignancies that developed in these patients were non-Hodgkin lymphoma (60%), Hodgkin disease (23%), and leukemia (6%) [5,6]. Approximately 20% of cancers are related to pathogens like Epstein–Barr virus, which can infect or reactivate in immunocompromised patients and plays a key role in carcinogenesis [4]. Immunity against EBV is primarily regulated by the adaptive cellular immune response, which involves major histocompatibility complex (MHC)-restricted cytotoxic cells; defects in these pathways can lead to harmful proliferation of EBV-infected B cells [6,7].

*CD137* (4-1BB), a member of the TNFR superfamily (*TNFRSF9*), has been identified as a costimulatory receptor that interacts with primed T lymphocytes [8]. Activated T cells express *CD137* on their surfaces, and *CD137* delivers costimulatory signals for T cell proliferation, apoptosis inhibition, and cytokine production when it is ligated by its native ligand (*CD137L*, 4-1BBL) or an agonist monoclonal antibody [9,10]. Additionally, the role of *CD137* in promoting the activation, proliferation, and survival of B cells has been investigated [11]. A *CD137* defect impairs T-cell co-stimulation and clearance of EBV-infected cells, resulting in persistent EBV infection and an increased risk of lymphoproliferative disease [12]. Experiments on knock-out mouse strains and observations obtained from individuals deficient in *CD137* have established that the *CD137–CD137L* interaction plays a primary role in antiviral cytotoxic T lymphocyte (CTL) responses by enhancing production of IFN-γ and perforin, which are essential mediators of the cytolytic activity of CD8+ T cells [13]. *CD137*, a protein associated with immune response regulation, is being explored as a potential avenue for cancer treatment [14,15]. Two main approaches have been investigated in clinical settings: the use of anti-*CD137* monoclonal antibodies and the incorporation of the *CD137* domain into chimeric antigen receptors (CARs) [10].

This article investigates a specific case of *CD137* deficiency, a rare IEI, and covers other cases reported in the literature.

## 2. Case Presentation

Written informed consent was obtained from a 21-year-old female patient initially referred to the hematology clinic due to progressive cervical lymphadenopathy that had persisted for several months and was associated with fever, intermittent night sweats, and weight loss. During childhood, she had recurrent skin abscesses, particularly affecting her back and legs, necessitating multiple hospitalizations for incision and drainage and intravenous antibiotics. Moreover, she had a history of recurrent otitis media. She had no medical history of pneumonia, meningitis, septic arthritis, or other deep-seated infections. Although she had a positive history of anemia, she did not have a documented history of failure to thrive or chronic diarrhea. Mild cognitive developmental delay was suspected. Prior to her presentation, she had no history of autoimmune or lymphoproliferative diseases, thrombocytopenia, or neutropenia before presentation.

Notably, three family members were diagnosed and treated for Hodgkin lymphoma; according to a recent report, they are currently in good health. Consanguinity is present in the family. The patient’s brother (who is 25 years old) was diagnosed at 9 years of age and underwent chemotherapy and an autologous stem cell transplant. The patient’s sister (who is 42 years old) was diagnosed in her mid-20s, underwent chemotherapy, and has been in remission since then. The patient’s nephew was diagnosed at 5 years of age; he was treated with chemotherapy followed by allogeneic stem cell transplant. Two paternal cousins were also diagnosed: one at the age of 13 years and the other in their 20s. The patient’s brother also has a history of facioscapulohumeral muscular dystrophy, which was diagnosed when he was 16 years old. Many other family members from the same tribe have been diagnosed with lymphoma.

Upon examination, the patient’s vital signs were found to be stable, and she exhibited a below-average-sized body weight. Lymphadenopathy was identified in her left axilla, left supraclavicular area, and cervical region with splenomegaly that was palpable approximately 3 cm below the costal margin.

## 3. Investigation

A computed tomography (CT) scan showed multifocal necrotic lymphadenopathy involving the left axillary, supraclavicular, mediastinal, and hilar regions, causing compression of mediastinal vessels, particularly the left pulmonary artery, with resultant left upper-lobe atelectasis and volume loss. An excisional biopsy of a cervical lymph node was performed, and histopathological analysis demonstrated architectural effacement by indistinct lymphoid nodules containing small lymphocytes, scattered Reed–Sternberg-like cells, and histiocytes, consistent with lymphocyte-rich classical Hodgkin lymphoma. Bone marrow examination showed hypercellular marrow with active trilineage hematopoiesis and no evidence of lymphoma involvement. Pre-chemotherapy blood tests revealed mild leukopenia (with a white blood cell count of 3.2 × 10^9^/L and a lymphocyte percentage of 29%). Lymphocyte subset analysis showed significant lymphopenia: the CD3+ (T cell) cell count was 404 cells/mm^3^ (reference range: 1200–2600 cells/mm^3^), the CD4+ (T-helper cell) count was 222 cells/mm^3^ (reference range: 650–1500 cells/mm^3^), the CD8+ (T-cytotoxic cell) count was 179 cells/mm^3^ (reference range: 370–1100 cells/mm^3^), the CD19+ (B cell) count was 45 cells/mm^3^ (reference range: 270–860 cells/mm^3^), and the CD3-CD16+CD56+ (NK cell) count was 111 cells/mm^3^ (reference range: 100–480 cells/mm^3^), with a CD4/CD8 ratio of 1.24%. Immunoglobulin levels were within normal ranges: antibody levels were as follows: IgG—14.20 g/L; IgA—1.98 g/L; and IgM—0.14 g/L.

Considering the potential for inherited immunodeficiency disorders and the limited availability of immunological functional studies, whole-exome sequencing was conducted, revealing a homozygous variant (c.325G>A p.(Gly 109Ser)) in the *TNFRSF9* gene, a result consistent with findings in the literature (Table 1). The patient exhibited blood serology that was positive for EBV, with negative EBV IgM levels but elevated EBV-IgG levels exceeding 750. Following the first cycle of chemotherapy, the patient exhibited low tetanus antibody levels (0.01 IU/mL) but normal pneumococcal antibody levels (11.9 IU/mL). Lymphocyte function tests were not available. Post-chemotherapy, a lymphocyte subset analysis demonstrated improvements in the lymphopenia: the levels of CD3+ cells (T cells), CD3+CD4+ cells (T-helper cells), CD3+CD8+ cells (T-cytotoxic cells), CD19+ cells (B cells), and CD3-CD16+CD56+ cells (NK cells) had increased to 705 cells/mm^3^, 487 cells/mm^3^, 200 cells/mm^3^, 35 cells/mm^3^, and 130 cells/mm^3^, respectively, with a CD4/CD8 ratio of 2.4%.

## 4. Treatment

The patient underwent six cycles of chemotherapy, comprising doxorubicin, vinblastine, dacarbazine, and brentuximab and achieved complete response at the end of therapy. Unfortunately, the patient exhibited clinical and histological evidence of cancer relapse 1.5 years later, prompting treatment with chemotherapy and allogeneic stem cell transplant. Afterward, the patient achieved remission before transplantation following seven cycles of salvage therapy with Nivolumab as per the protocol for refractory and persistent disease. Hematopoietic stem cell transplantation was performed subsequently, with her brother, a full HLA-matched donor who is heterozygous for the *TNFR95* mutation, serving as the donor.

A myeloablative conditioning regimen consisting of fludarabine and melphalan was administered. Graft-versus-host disease (GVHD) prophylaxis included post-transplant cyclophosphamide, cyclosporine for 3 months, and Mycophenolate mofetil for 30 days.

As of now (two year post transplant), the patient is no longer undergoing immunosuppression therapy with no active GVHD and no recurrent infections. PET and other radiological scans performed a few times post-transplant confirmed that she remains in complete remission. EBV PCR repeated multiple times from blood and was negative. 

## 5. Discussion and Literature Review

Our patient’s presentation and diagnosis align with several cases recently documented in the literature. These cases have been thoroughly discussed and investigated, shedding light on the clinical manifestations and immune abnormalities linked to *CD137* deficiency. The focus of these studies includes exploring the genetic basis, immune cell phenotypes, and potential implications for therapeutic interventions.

Alosaimi et al. [16] identified two unrelated patients who shared a homozygous G109S missense mutation in the 4-1BB gene (*TNFRSF9*). This mutation led to the complete absence of 4-1BB surface expression and prevented the mutated protein from binding to its ligand, 4-1BBL. Like our patient, both patients had a history of recurrent sinopulmonary infections, persistent EBV viremia, and EBV-driven lymphoproliferation [16].

Patient 1 experienced recurrent sinopulmonary infections and bronchiectasis at the age of 3, which improved with intravenous gamma-globulin replacement therapy. By 5 years of age, she developed generalized lymphadenopathy and EBV viremia, which later progressed to hemophagocytic lymphohistiocytosis, representing an earlier presentation of lymphoproliferative disease relative to our patient. She underwent chemotherapy and received anti-CD20 monoclonal antibody (rituximab) treatment in preparation for hematopoietic stem cell transplantation from an HLA-matched sibling. Patient 2 had similarly recurrent sinopulmonary infections, alongside generalized lymphadenopathy, increased IgG and IgA levels, and low IgM levels. He was diagnosed with EBV-positive Hodgkin disease and underwent chemotherapy and rituximab treatment that resulted in decreased EBV viremia and lymphadenopathy; however, the disease relapsed after 1 year, a phenomenon comparable to the rapid relapsing pattern exhibited by our patient (Table 1) [16].

Whole-exome sequencing identified a homozygous missense mutation in the 4-1BB gene at position 109 (G109S) that abolished 4-1BB surface expression and ligand binding; immuno-blotting confirmed the absence of mutant protein expression. PHA stimulation induced 4-1BB surface expression on CD8+ T-cells from control subjects but not from the patients. Several immunological studies were conducted, indicating impaired CD8+ T-cell expansion and cytotoxic function, reduced IFN-γ and perforin expression, and reduced EBV-specific cytotoxic activity relative to healthy donors [13,16]. This impairment in EBV-specific immunity manifested as recurrent EBV viremia and lymphoproliferation in these patients. Mitochondrial function, which is crucial for activated T cells and their cytotoxic function, was negatively affected by 4-1BB deficiency in T cells, contributing to decreased cytotoxicity [16,17]. This research underscores a novel immunodeficiency and highlights the critical role the 4-1BB receptor plays in protecting the host against EBV infections. In immunodeficient patients, such as those in this study, the lack of functional 4-1BB renders them susceptible to chronic EBV infection and its associated complications [12,16].

Somekh et al. [18] explored the impact of *CD137* deficiency on the immune system and its correlation with elevated susceptibility to lymphoma development [18,19,20]. *CD137*, a member of the tumor necrosis factor receptor superfamily, plays a crucial role in immune regulation and co-stimulation [8]. Their study involved clinical observations, genetic analysis, and laboratory experiments and focused on patients with recurrent infections, immune dysregulation, and an increased predisposition to lymphomagenesis due to *CD137* deficiency [18].

The clinical observations in the aforementioned study were based on four patients from unrelated families, including three with consanguineous backgrounds [18]. All patients exhibited recurrent infections, signs of autoimmunity, abnormal immunoglobulin levels, and notable organ enlargement (Table 1). Strikingly, two of these patients manifested lymphomas, establishing a clear association between *CD137* deficiency and susceptibility to lymphomagenesis. Genetic analysis revealed distinct homozygous variants in *TNFRSF9,* leading to a significant reduction in or the complete absence of *CD137* expression on activated T, B, and NK cells. Interestingly, in this study, the author noted that one patient’s sibling was homozygous for the same *TNFRSF9* mutations but did not exhibit overt clinical symptoms. One possible explanation is incomplete penetrance, a common feature of pathogenic immune system mutations in which some individuals carrying the pathogenic variant express the associated disease trait while others do not [21].

Examination of immune cell phenotypes in these patients revealed a spectrum of abnormalities, including altered B cell-population proportions and diminished counts of memory B cells, plasmablasts, and NK cells. Functional analysis demonstrated impaired T cell-proliferation responses across all patients. Patient 4 had near-normal T cell activation, and a minor T cell proliferation defect indicated that the *CD137* pathway was affected but less severely (in terms of function) in this individual. Further analysis of TCR repertoires unveiled clonal expansion and reduced diversity in these three patients. Additional observations included lower Treg frequencies and diminished capacities for EBV-specific-T cell cytotoxicity, suggesting *CD137*-deficient individuals are more susceptible to EBV-associated lymphomagenesis [12,18]. Significantly, in *CD137*-deficient patients, the authors found B cell defects that involved impaired activation, proliferation, compromised class-switch recombination, and impaired maturation of memory B cells and plasmablasts—outcomes that were directly attributed to the complete lack of *CD137* signaling [11,18].

Fournier et al. [22] explored the links between inherited *TNFSF9* deficiency, broad EBV infection, and EBV-positive smooth muscle tumors. Their research concerned an 18-year-old female with a *TNFSF9* deficiency that impairs *CD137* function, making her susceptible to uncontrolled EBV infections. Whole-exome sequencing was conducted to examine the genetic etiology of the patients’ conditions, with a focus on vulnerability to EBV infection. A 3 Mb de novo heterozygous 22q11.2 deletion related to DiGeorge syndrome was identified. This finding explained the patient’s intellectual and growth issues, although the patient did not display common DiGeorge symptoms. A homozygous mutation was identified in the *TNFSF9* gene encoding the *CD137L* protein, a T cell co-stimulatory molecule and ligand of *CD137*, and this mutation is linked to EBV susceptibility [16,17,18]. The V140G variation in *TNFSF9* was identified as a significant genetic candidate in terms of potentially explaining the patient’s EBV susceptibility (Table 1). In summary, the study by Fournier et al. explored the genetic mutations responsible for inherited *TNFSF9* deficiency, which leads to EBV infections and EBV-positive smooth muscle tumors. It also revealed the clinical manifestations and immune cell abnormalities associated with this condition [22]. The findings demonstrated *CD137’s* role in immune system regulation and its potential as a target for immunotherapy, especially in malignancies [14,22].

Shen et al. [23] reported an unusual case of severe EBV-associated lymphoproliferative disease secondary to *CD137* deficiency. The patient’s medical history, marked by recurrent bacterial and viral infections indicative of an impaired immune system, revealed severe symptoms associated with EBV-associated lymphoproliferative disease (Table 1). Common symptoms in primary immunodeficiency (IEI) cases, such as hepatomegaly, splenomegaly, and lymphadenopathy, were evident along with signs of autoimmunity, including hemolytic anemia [1,2]. This case shares some similarities with our patient and other previously reported *CD137* deficiency patients, although with additional manifestations more commonly observed in primary immunodeficiency disorders (PIDs) [1,23].

Comprehensive genetic analysis investigating the underlying cause of the patient’s condition identified two novel biallelic mutations in the *TNFRSF9* gene encoding *CD137* [23]. In particular, each allele bore distinct mutations, making this a particularly rare case. The presence of biallelic mutations in *TNFRSF9* allowed for a conclusive diagnosis of *CD137* deficiency, shedding light on affected individuals’ susceptibility to severe EBV-associated lymphoproliferative disease [12,23]. The study further explored the impact of compound heterozygous *TNFRSF9* mutations on *CD137* deficiency. The mutations were found to diminish or abolish *CD137* expression on activated CD4+ T cells, CD8+ T cells, B cells, and NK cells, indicating a loss-of-function phenotype [11,13,23]. Notably, cells from heterozygous parents showed a partial or substantial decrease in *CD137* expression compared with cells from healthy donors, suggesting that *CD137* deficiency was determined in a gene-oriented dose-dependent manner, wherein *CD137* expression decreases with a reduction in the number of functional gene copies [23,24].

Although the patient’s CD4+ T cells exhibited reduced *CD137* expression, they unexpectedly maintained normal activation, suggesting possible compensation through alternative costimulatory pathways [17,19]. Conversely, CD8+ T cells exhibited significant reductions in the expression of IFN-γ, TNF-α, perforin, and granzyme B, pointing to a compromised cytotoxic response. NK cells, however, showed normal cytotoxicity and degranulation. This study highlights the intricate role of *CD137* in immune homeostasis, showcasing dysregulation across both T cells and B cells in the patient [23,25]. The two novel biallelic *TNFRSF9* mutations found in this patient underscore the genetic complexity of *CD137* deficiency, helping to foster a deeper understanding of this condition and reveal potential avenues for targeted therapies [10,25,26]. This study illuminated a rare case of *CD137* deficiency, emphasizing the critical role of *CD137* in the immune response to viral infections and providing valuable insights into the associated immune system dysregulation, T cell dysfunction, and B cell abnormalities [11,13,25].

Rodriguez et al. [27] reported a patient with recurrent infections early in life who later developed striking clinical features of EBV-associated T-cell lymphoproliferative disease. Histologic analysis revealed liver infiltration by EBV-infected T cells. The patient’s levels of EBV viremia remained high despite treatment with an anti-CD20 monoclonal antibody. Around the age of 14 years, he developed fatal hemophagocytic lymphohistiocytosis. His immunological profile was notable for fluctuating lymphopenia, decreased levels of naïve CD8+ T cells, an increased proportion of memory CD8+ T cells, and a lack of circulating memory and class-switched B cells. Levels of NK cells, invariant NKT cells, and mucosal-associated invariant T cells were also reduced. Given his presentation, immunodeficiency was suspected. Genetic testing was conducted on both the patient and his asymptomatic sister, who was afflicted with isolated persistent EBV viremia. The results revealed a significant homozygous variation in *TNFRSF9* (Table 1). Interestingly, despite persistent EBV viremia and circulating EBV-infected T cells, the patient’s sister had not developed clinical symptoms, potentially suggesting incomplete penetrance. However, as the patient’s clinical phenotype was different from that of his asymptomatic sister, the authors suggested he might have been a carrier of an additional genetic factor [27,28]. Thus, further genetic analysis was conducted, identifying a significant missense biallelic mutation in *PIK3CD*, as confirmed by Sanger sequencing, and this mutation was shown to follow an autosomal recessive inheritance pattern [27]. *PIK3CD* deficiency has recently been identified in four patients presenting with immunodeficiency; however, the functional impact of the mutation was not investigated in these cases [27,29,30,31].

Zhao et al. [32] recently described a four-year-old boy born to consanguineous Chinese parents who initially presented with recurrent infections, conjunctivitis, and an abdominal mass. He was diagnosed with Burkitt’s lymphoma and treated according to the SCCCG-BL-2017 protocol, which included corticosteroids, cyclophosphamide, vincristine, cytarabine, methotrexate, and doxorubicin. He later developed recurrent respiratory and ear infections, hepatosplenomegaly, and generalized lymphadenopathy. Laboratory evaluation demonstrated chronic Epstein–Barr virus infection. Owing to persistent infections and EBV viremia, he ultimately underwent hematopoietic stem-cell transplantation. Notably, his 16-year-old sibling had previously died from nasopharyngeal carcinoma. A genetic test identified a homozygous missense variant (c.359G>C; p.C120S) in the *TNFRSF9* gene (*NM_001561.5*) (Table 1). The variant exhibited an autosomal recessive inheritance pattern, with one mutant allele inherited from each parent. Additionally, in a manner similar to the findings reported by Alosaimi et al., functional immunologic assessment of the patient’s CD8+ T cells demonstrated reduced IFN-γ release compared with the mother, suggesting impaired CD8+ T-cell function. Furthermore, the patient had reduced levels of memory B cells (CD19+ CD27+) with impairment of B-cell maturation and differentiation [32,33,34]. Zhao et al. [32] investigated the potential effect of this mutation on NF-kB signaling activation. The study revealed a reduced level of activated Protein kinase B (PKB), which plays a major role in cell survival, proliferation, transcription, and migration [29,32]. These results underline the crucial role of *CD137* in lymphocyte and general immune cell activity and are in line with previously documented cases and the literature [32,35,36].

In conclusion, these cases highlight the vital function of *CD137* in immunological regulation and its consequences for human health [13,14,16,17,20,21]. The distinct and unique nature of *CD137* deficiency is revealed by the genetic mutations discovered in *TNFRSF9*, advancing our knowledge of primary immunodeficiency diseases. The clinical signs demonstrate the severe effects of *CD137* loss on immunological function, including increased susceptibility to lymphomagenesis and recurrent infections [4,11,12,13]. Additionally, these 12 cases with varying presentations indicate the complexity of *CD137* as a crucial regulator of immune homeostasis. Additionally, *CD137* (4-1BB), a costimulatory receptor expressed on activated immune cells, is increasingly attracting interest as a target for cancer immunotherapy due to its ability to augment T-cell activation, proliferation, cytokine production, and antitumor activity [10,14,15]. Specifically, two main approaches have been investigated in clinical settings: the use of anti-*CD137* monoclonal antibodies and the incorporation of the *CD137* domain into chimeric antigen receptors (CARs) [10].

**Table 1:** Clinical features and genetic mutations [16,18,22,23,27,32].

Study	Patient No.	Patient Characteristics	Clinical Presentation	EBV State	Treatment	Mutation
Alosaimi et al. [16]	Patient 1	Saudi Female Consanguineous family	3 years old: Recurrent sinopulmonary infections; Bronchiectasis; Pneumococcal septicemia; Hypogammaglobulinemia. 5 years old: EBV viremia; Pancytopenia; EBV+ B-cell LPD; Hemophagocytic lymphohistiocytosis.	EBV viremia Cervical lymph node: multiple clusters of CD20+ B cells positive for EBV-encoded RNA	IVIG Anti-CD20 mAb (rituximab) HSCT	Homozygous TNFRSF9 variant (NM_001561:c.325G>A: p.Gly109Ser)
	Patient 2	Saudi Male Consanguineous family	6 years old: Recurrent sinopulmonary infections; Lymphadenopathy; Splenomegaly; EBV viremia; EBV-positive Hodgkin’s disease; Progressed to diffuse large B-cell lymphoma.	EBV viremia Lymph node: positive for EBV-encoded RNA (EBER-positive)	IVIG Chemotherapy Anti-CD20 mAb (rituximab)	Homozygous TNFRSF9 variant (NM_001561:c.325G>A: p.Gly109Ser)
Somekh et al. [18]	Patient 3	Turkish Male Consanguineous family	2 years old: Recurrent ear infections; Hepatosplenomegaly; Hypogammaglobulinemia; EBV+ Burkitt’s lymphoma; EBV viremia.	EBV viremia EBV+ Burkitt’s lymphoma	Chemotherapy Anti-CD20 mAb (rituximab) IVIG Antibiotics	Homozygous TNFRSF9 variant (c.1_545+1716del)
	Patient 4	Palestinian Male Consanguineous family	3 years old: Recurrent pneumonia 6 years old: Autoimmune lymphoproliferative syndrome-like disease; Hepatosplenomegaly; Lymphadenopathy; Autoimmunity (i.e., AIHA, ITP, and ANA positivity); EBV viremia.	EBV viremia Lymph node: EBV-related LPD with a monoclonal T-cell population (EBER-positive)	Sirolimus Glucocorticoids CellCept for autoimmunity Antibiotic prophylaxis	Homozygous TNFRSF9 variant (NM_001561.5: c.452C>T; p.Thr151Met)
	Patient 5	Turkish Male Consanguineous family	6 years old: Herpes labialis; Lower-respiratory-tract infection; Hepatosplenomegaly; Lymphadenopathy. 8 years old: Pneumonia; Recurrent tonsillitis; Otitis media and chronic suppurative otitis media; Atopic dermatitis and xerosis; Positive direct Coombs test; Hypergammaglobulinemia; EBV viremia. 10 years old: EBV-positive Hodgkin’s lymphoma.	EBV viremia Lymph node: EBER-positive	Chemotherapy IVIG Amoxicillin prophylaxis	Homozygous TNFRSF9 variant: (NM_001561.5: c.101 −1G>A)
	Patient 6	Colombian Male Non-consanguineous family	Since the age of 8 years: Recurrent otitis media; Sinusitis; EBV viremia. Between 20 and 22 years old: Recurrent pneumonia; Common variable immunodeficiency; Granulomatous pleuropneumonia; Helicobacter pylori erythematous gastritis; Chronic sinusitis.	EBV viremia	SCIG	Homozygous TNFRSF9 variant: (NM_001561.5: c.100 +1G>A)
Fournier et al. [22]	Patient 7	Moroccan Female Consanguineous family Presented at 18 years old	Since the age of 9 years old: Disseminated EBV+ SMTs that grew slowly and were not invasive; One large compressive proximal bronchial lesion and severely damaged lung parenchyma associated with recurrent bacterial infections Adenopathy; EBV viremia; No other symptoms of immune deficiency; Other non-hematological clinical features: delayed milestones, mild intellectual deficit, growth retardation, severe amyotrophy, and palatine insufficiency.	EBV viremia Spleen biopsy: EBV+ SMT cells with EBV-infected B and T cells in the red pulp	Surgery: - Total splenectomy - Removal of accessible tumors Chemotherapy	Homozygous TNFRSF9 variant: (g.9:6534730T>G, the c.419T>G) De novo heterozygous 22q11.2 deletion
Shen et al. [23]	Patient 8	Chinese Female Non-consanguineous family	16 years old: 2-month history of intermittent low-grade fever and tonsillitis; EBV+ B-cell LPD; Cytopenia; EBV viremia and chronic active EBV infection; Multiorgan failure; Sinopulmonary infections; Transformation into malignant lymphoma; Severe lung infections, including *Pseudomonas fluorescens*, *Candida albicans*, *Aspergillus niger*, and EBV.	EBV viremia Tonsil biopsy: EBV+ LPD grade 1 Left cervical lymph node: EBV+ LPD grade 2	Antifungal, antibiotic, and antiviral therapy Dexamethasone Bortezomib HSCT	Two novel compound TNFRSF9 gene heterozygous mutations: *A TNFRSF9 splicing mutation* (NM_001561.5:c.208 + 1−>AT) from healthy father *A TNFRSF9 missense mutation* (NM_001561.5:c.452C>A, p. T151K) from healthy mother
Rodriguez et al. [27]	Patient 9	Pakistan Male Consanguineous family	4 months old: Recurrent upper respiratory tract infections; Recurrent skin infections. 9 years 10 months old: Persistent fever; Weight loss; Hepatosplenomegaly; Recurrent lymphadenopathies; EBV viremia. 14 years old: Fatal hemophagocytic lymphohistiocytosis.	EBV viremia Liver biopsy: EBER probe was positive in CD3+ cells, while most CD20+ B cells were negative	Anti-CD20 mAb (rituximab) Antibiotic	Homozygous TNFRSF9 variant: (NM_001561.5: c.170DelG) Biallelic mutation in PIK3CD (NM_005026.3 c.2462G>A)
	Patient 10	Pakistan Female Consanguineous family Sibling of Patient 9	6 years old: EBV viremia. 8 years old: Asymptomatic persistent high EBV viremia.	EBV viremia	Anti-CD20 mAb (rituximab)	Homozygous TNFRSF9 variant: (NM_001561.5: c.170DelG)
Zhao et al. [32]	Patient 11	Chinese Male Consanguineous family	4 years old: Recurrent respiratory infections; Conjunctivitis; Burkitt’s lymphoma. Between 4 and 8 years old: Recurrent respiratory infections; Ear Infections; Hepatosplenomegaly; Lymphadenopathy; Chronic EBV infection. 16-year-old sibling who died due to nasopharyngeal carcinoma (NPC)	EBV viremia	Chemotherapy SCCCG-BL-2017 regimen: “Cyclophosphamide, vincristine, cytarabine, methotrexate, doxorubicin.” IVIG Antibiotic HSCT	Homozygous Missense Variant TNFRSF9 gene (NM_001561.5) (c.359G>C, p.C120S).
Algrafi et al. (present case)	Patient 12	Saudi Female Consanguineous family Presented at 21 years old	From age 3 years old: Recurrent skin abscess; Recurrent otitis media. 21 years old: Lymphadenopathy; Classical Hodgkin’s lymphoma. 23 years old: Recurrence of Hodgkin’s lymphoma.	EBV viremia	Antibiotic Surgical drainage Chemotherapy HSCT	Homozygous TNFRSF9 variant: (c.325G>A p.(Gly 109Ser)

IVIG: intravenous immunoglobulin, SCIG: subcutaneous immunoglobulin, EBV: Epstein–Barr virus, AIHA: autoimmune hemolytic anemia, ITP: immune thrombocytopenia, EBER: EBV-encoded small RNAs, SMTs: smooth muscle tumors, HSCT: hematopoietic stem cell transplantation, LPD: lymphoproliferative disease, mAb: monoclonal antibody.

## Data Availability

The data presented in this study are available on request from the corresponding author.

## References

[1] McCusker C., Upton J., Warrington R. (2018). Primary immunodeficiency. Allergy Asthma Clin. Immunol..

[2] Poli M.C., Aksentijevich I., Bousfiha A.A., Cunningham-Rundles C., Hambleton S., Klein C., Morio T., Picard C., Puel A., Rezaei N. (2025). Human inborn errors of immunity: 2024 update on the classification from the International Union of Immunological Societies Expert Committee. J. Hum. Immun..

[3] Abolhassani H., Azizi G., Sharifi L., Yazdani R., Mohsenzadegan M., Delavari S., Sohani M., Shirmast P., Chavoshzadeh Z., Mahdaviani S.A. (2020). Global systematic review of primary immunodeficiency registries. Expert. Rev. Clin. Immunol..

[4] Salavoura K., Kolialexi A., Tsangaris G., Mavrou A. (2008). Development of cancer in patients with primary immunodeficiencies. Anticancer Res..

[5] Frizzera G., Rosai J., Dehner L.P., Spector B.D., Kersey J.H. (1980). Lymphoreticular disorders in primary immunodeficiencies: New findings based on an up-to-date histologic classification of 35 cases. Cancer.

[6] Mueller B.U., Pizzo P.A. (1995). Cancer in children with primary or secondary immunodeficiencies. J. Pediatr..

[7] Rodriguez-Abreu D., Bordoni A., Zucca E. (2007). Epidemiology of hematological malignancies. Ann. Oncol..

[8] Kwon B.S., Weissman S.M. (1989). cDNA sequences of two inducible T-cell genes. Proc. Natl. Acad. Sci. USA.

[9] Otano I., Azpilikueta A., Glez-Vaz J., Alvarez M., Medina-Echeverz J., Cortés-Domínguez I., Ortiz-De-Solorzano C., Ellmark P., Fritzell S., Hernandez-Hoyos G. (2021). CD137 (4-1BB) costimulation of CD8+ T cells is more potent when provided in cis than in trans with respect to CD3-TCR stimulation. Nat. Commun..

[10] Etxeberria I., Glez-Vaz J., Teijeira Á., Melero I. (2020). New emerging targets in cancer immunotherapy: CD137/4-1BB costimulatory axis. ESMO Open.

[11] Zhang X., Voskens C.J., Sallin M., Maniar A., Montes C.L., Zhang Y., Lin W., Li G., Burch E., Tan M. (2010). CD137 promotes proliferation and survival of human B cells. J. Immunol..

[12] Latour S., Fischer A. (2019). Signaling pathways involved in the T-cell-mediated immunity against Epstein-Barr virus: Lessons from genetic diseases. Immunol. Rev..

[13] Wen T., Bukczynski J., Watts T.H. (2002). 4-1BB ligand-mediated costimulation of human T cells induces CD4 and CD8 T cell expansion, cytokine production, and the development of cytolytic effector function. J. Immunol..

[14] Liu G., Luo P. (2023). Targeting CD137 (4-1BB) towards improved safety and efficacy for cancer immunotherapy. Front. Immunol..

[15] Claus C., Ferrara-Koller C., Klein C. (2023). The emerging landscape of novel 4-1BB (CD137) agonistic drugs for cancer immunotherapy. mAbs.

[16] Alosaimi M.F., Hoenig M., Jaber F., Platt C.D., Jones J., Wallace J., Debatin K.-M., Schulz A., Jacobsen E., Möller P. (2019). Immunodeficiency and EBV-induced lymphoproliferation caused by 4-1BB deficiency. J. Allergy Clin. Immunol..

[17] Teijeira A., Labiano S., Garasa S., Etxeberria I., Santamaría E., Rouzaut A., Enamorado M., Azpilikueta A., Inoges S., Bolaños E. (2018). Mitochondrial Morphological and Functional Reprogramming Following CD137 (4-1BB) Costimulation. Cancer Immunol. Res..

[18] Somekh I., Thian M., Medgyesi D., Gülez N., Magg T., Duque A.G., Stauber T., Lev A., Genel F., Unal E. (2019). CD137 deficiency causes immune dysregulation with predisposition to lymphomagenesis. Blood.

[19] Herber M., Mertz P., Dieudonné Y., Guffroy B., Jung S., Gies V., Korganow A.-S., Guffroy A. (2020). Primary immunodeficiencies and lymphoma: A systematic review of literature. Leuk. Lymphoma.

[20] Bin Riaz I., Faridi W., Patnaik M.M., Abraham R.S. (2019). A systematic review on predisposition to lymphoid (B and T cell) neoplasias in patients with primary immunodeficiencies and immune dysregulatory disorders (inborn errors of immunity). Front. Immunol..

[21] Gruber C., Bogunovic D. (2020). Incomplete penetrance in primary immunodeficiency: A skeleton in the closet. Hum. Genet..

[22] Fournier B., Hoshino A., Bruneau J. (2022). Inherited TNFSF9 deficiency causes broad Epstein-Barr virus infection with EBV+ smooth muscle tumors. J. Exp. Med..

[23] Shen K., Wang J., Zhou K., Mu W., Zhang M., Deng X., Cai H., Zhang W., Huang W., Xiao M. (2023). CD137 deficiency because of two novel biallelic TNFRSF9 mutations in a patient presenting with severe EBV-associated lymphoproliferative disease. Clin. Trans. Immunol..

[24] Rice A.M., McLysaght A. (2017). Dosage-sensitive genes in evolution and disease. BMC Biol..

[25] Frauwirth K.A., Thompson C.B. (2002). Activation and inhibition of lymphocytes by costimulation. J. Clin. Investig..

[26] Vinay D.S., Kwon B.S. (2014). 4-1BB (CD137), an inducible costimulatory receptor, as a specific target for cancer therapy. BMB Rep..

[27] Rodriguez R., Fournier B., Cordeiro D.J., Winter S., Izawa K., Martin E., Boutboul D., Lenoir C., Fraitag S., Kracker S. (2019). Concomitant PIK3CD and TNFRSF9 deficiencies cause chronic active Epstein-Barr virus infection of T cells. J. Exp. Med..

[28] Similuk M., Kuijpers T. (2023). Nature and nurture: Understanding phenotypic variation in inborn errors of immunity. Front. Cell. Infect. Microbiol..

[29] Sogkas G., Fedchenko M., Dhingra A., Jablonka A., Schmidt R.E., Atschekzei F. (2018). Primary immunodeficiency disorder caused by phosphoinositide 3-kinase δ deficiency. J. Allergy Clin. Immunol..

[30] Cohen S.B., Bainter W., Johnson J.L., Lin T.-Y., Wong J.C., Wallace J.G., Jones J., Qureshi S., Mir F., Qamar F. (2019). Human primary immunodeficiency caused by expression of a kinase-dead p110δ mutant. J. Allergy Clin. Immunol..

[31] Swan D.J., Aschenbrenner D., Lamb C.A., Chakraborty K., Clark J., Pandey S., Engelhardt K.R., Chen R., Cavounidis A., Ding Y. (2019). Immunodeficiency, autoimmune thrombocytopenia and enterocolitis caused by autosomal recessive deficiency of PIK3CD-encoded phosphoinositide3-kinase δ. Haematologica.

[32] Zhao P., Chen K., Yang L., Wan C., Zhang L., Luo S., He X. (2025). Clinical and functional characterization of a novel TNFRSF9 variant causing immune dysregulation with predisposition to EBV-driven lymphomagenesis. Front. Immunol..

[33] Middendorp S., Xiao Y., Song J.-Y., Peperzak V., Krijger P.H.L., Jacobs H., Borst J. (2009). Mice deficient for CD137 ligand are predisposed to develop germinal center-derived B-cell lymphoma. Blood.

[34] Pauly S., Broll K., Wittmann M., Giegerich G., Schwarz H. (2002). CD137 is expressed by follicular dendritic cells and costimulates B lymphocyte activation in germinal centers. J. Leukoc. Biol..

[35] Jones R.G., Saibil S.D., Pun J.M., Elford A.R., Bonnard M., Pellegrini M., Arya S., Parsons M.E., Krawczyk C.M., Gerondakis S. (2005). NF-kappaB couples protein kinase B/Akt signaling to distinct survival pathways and the regulation of lymphocyte homeostasis in vivo. J. Immunol..

[36] Alfaro C., Echeveste J.I., Rodriguez-Ruiz M.E., Solorzano J.L., Perez-Gracia J.L., Idoate M.A., Lopez-Picazo J.M., Sanchez-Paulete A.R., Labiano S., Rouzaut A. (2015). Functional expression of CD137 (4-1BB) on T helper follicular cells. Oncoimmunology.

